# Seed Priming with ZnO and Fe_3_O_4_ Nanoparticles Alleviate the Lead Toxicity in *Basella alba* L. through Reduced Lead Uptake and Regulation of ROS

**DOI:** 10.3390/plants11172227

**Published:** 2022-08-28

**Authors:** Nakul Gupta, Prabhakar Mohan Singh, Vidya Sagar, Alok Pandya, Manimurugan Chinnappa, Rajesh Kumar, Anant Bahadur

**Affiliations:** 1 ICAR-Indian Institute of Vegetable Research, PB-01, Po-Jakhini (Sahanshahpur), Varanasi 221305, Uttar Pradesh, India; 2 Department of Engineering & Physical Sciences, Institute of Advanced Research, Koba Institutional Area, Gandhinagar 382426, Gujarat, India; 3 ICAR-Indian Institute of Oilseeds Research, Rajendranagar, Hyderabad 500030, Telangana, India

**Keywords:** ZnO nanoparticles, Fe_3_O_4_ nanoparticles, lead toxicity, *Basella alba*, ROS and antioxidants

## Abstract

The increased lead (Pb) content in the environment has an impact on all living beings, including plant growth and quality. The present study aims to investigate the protective roles of zinc (Zn)- and iron (Fe)- nanoparticles (NPs) in alleviating stress symptoms caused by lead (Pb) exposure in *Basella alba* seedlings. For this purpose, 15 different treatment combinations of seed priming with two NPs at 0 and 200 mg L^−1^, and five Pb levels (0, 4, 8, 15, 20 mM) were chosen. Pb stress (20 mM) was found to reduce seed germination by 72.8% and seedling growth, particularly root length, by 92% when compared to the control. Under different Pb concentrations, seed priming with ZnNPs (200 mg L^−1^) and FeNPs (200 mg L^−1^) increased seed germination by 34.7% and 54.9%, respectively, and root length by 152.9% and 252.9%, respectively. In 20 mM Pb stress, NPs primed seedling showed decrease in Pb content by 33.7% with ZnNPs and 32.6% with FeNPs. Increased Pb stress resulted in increased reactive oxygen species (ROS) generation (H_2_O_2_) and lipid peroxidation (MDA) compared to non-Pb stressed seedlings. However, increased antioxidants in the NPs treatments such as SOD, CAT, POD and proline content, scavenged these ROS. Considering all the parameters under study, priming alleviated Pb stress in the following order: FeNPs > ZnNPs > hydropriming > control. To summarise, seed priming with Zn- and Fe-NPs has the potential to alleviate Pb toxicity via reduced Pb uptake, ROS generation and lipid peroxidation as well as increased proline content and activation of antioxidant enzymatic system.

## 1. Introduction

Heavy metal contamination in soil, such as lead (Pb), mercury (Hg), barium (Ba) and cadmium (Cd), is a serious global concern caused by different anthropogenic activities such as untreated sewage water and industrialization [1,2]. The non-degradable nature of Pb makes it a potentially toxic element to plant growth and humans [3,4], as Pb can replace cationic ions of similar size such as Ca^2+^, Mg^2+^, Fe^2+^ and Na^+^, disrupting a cell’s biological metabolism [5]. Food grown in Pb contaminated soil is the primary source of human exposure to Pb, because it enters the food chain via roots absorption [6]. Consumption of these foods in excess of the acceptable weekly Pb intake limit of ~25 μg kg^−1^ of human body weight has an adverse effects on the vital organs [7]. Pb concentrations in southern Indian soil ranges from 36.6–71.5 mg kg^−1^ and in North Indian soil ranges from 21.0–40.78 mg kg^−1^, with an average of 52.57 mg kg^−1^ and 32.14 mg kg^−1^, respectively [8]. Pb concentrations in plants greater than 2 mg kg^−1^ can impair nutrient uptake, water relations and generation of reactive oxygen species (ROS), resulting in reduced photosynthesis, high cell death, and ultimately reduced plant yield [9]. Effect of Pb begins with the onset of germination process [10,11] and it retards radicle growth by decreasing the enzymes involved in the reserve metabolism [12].

Seed priming with nanoparticles (NPs) is a novel feasible approach to protect the plant against heavy metal toxicity, because a small quantity of chemical is used [13,14], whereas other methods, such as soil application, require a higher dose of amendment due to low nutrient use efficiency [15]. However, only a few studies have been conducted to determine the efficacy of NPs seed priming in providing Pb stress tolerance. NPs have certain important physio-chemical properties due to their small particle size (1 to 100 nm), such as higher surface area, high reactivity, high solubility, high penetration capacity, and high surface: volume ratio [16], making them ideal for use as seed priming agent. Numerous studies have shown that NPs have dose-dependent biological effects (both positive and negative) not only on plant growth and yield [14,17,18,19] but also against the biotic and abiotic stresses in plants [20].

Iron and zinc are consecutively the first and second most abundant transition metals in nature, respectively, and are essential for various biological and metabolic processes in plants [21] by acting as a cofactors in over 300 enzymes [22]. These nutrients may be applied directly to the soil, through foliar application, or added as seed treatment such as priming and coating in crop plants. Seed being a small delicate living entity, it is more vulnerable to Pb stress. Faster and more uniform seed germination, as well as the establishment of vigorous crop are critical under Pb stress condition, which is dependent on seed germination and vigour *per se*. Therefore, it was hypothesized that seed priming with NPs (ZnO and Fe_3_O_4_) could improve the seed germination and early seedling vigour by improving the physio-biochemical performance of seed under Pb stress. Given that leafy and root vegetables are more prone to heavy metal accumulation and are used as indicator plants for heavy metal stress [23,24,25,26], a leafy vegetable *Basella alba* was selected as target crop for studying the effect of Pb on germinating seedlings. *B. alba,* a member of the Basellaceae family is commonly known as Indian spinach or Malabar spinach and is grown for its high nutritional (proteins, fat, vitamins, such as A, C, E, K, folic acid, riboflavin, niacin, thiamine and minerals) and pharmaceutical values (treatment of dysentery, diarrhoea, anaemia, cancer, and wound healing) [27,28]. The following objectives were pursued in this study: (1) to investigate the effect of Pb toxicity on seeds and seedlings of *B. alba* (2) to determine whether seed priming with ZnO- and Fe_3_O_4_-NPs can alleviate Pb toxicity in *B. alba* seedlings and (3) to understand the underlying physiochemical changes in order to alleviate the Pb toxicity in *B. alba* seedlings.

## 2. Materials and Methods

### 2.1. Synthesis and Characterization of ZnO- and Fe_3_O_4_-NPs

ZnO- and Fe_3_O_4_-NPs were synthesized and characterized at Department of Engineering and Physical Sciences, Institute of Advanced Research, Gandhinagar, Gujarat, India. Both the NPs were synthesized with minor modification in the method described by Vallabani et al. [29].

ZnO-NPs

Citrate-coated ZnO-NPs was synthesized by dissolving 2.5 g ZnSO_4_ in 50 mL MilliQ water. The solution was heated at 70 °C for 30 min with continuous bubbling of nitrogen. Thereafter, the solution was further heated with continuous stirring at 70 °C for 2 h and later cooled to room temperature. Further the solution was centrifuged at 3000 rpm for 10 min followed by collection of the supernatant. The final concentration of ZnO- NPs in the supernatants was 1 mg mL^−1^.

Fe_3_O_4_-NPs

Citrate-coated Fe_3_O_4_-NPs was synthesized by making a solution of 2.5 g FeCl_3_.6H_2_O and 1.2 g FeSO_4_.7H_2_O in 60 mL MilliQ water. The solution was heated at 70 °C for 30 min with continuous bubbling of nitrogen. Thereafter, the solution was further heated with continuous stirring at 70 °C for 2 h and later cooled to room temperature. Further the solution was centrifuged at 3000 rpm for 10 min followed by collection of the supernatant. The final concentration of Fe_3_O_4_-NPs in the supernatants was 2 mg mL^−1^.

The suspensions of both the NPs in water were incubated for 15 to 20 min in an ultrasonic bath to disperse the particles. Further, this sample was analysed by Transmission Electron Microscope (JEM1400 plus, JEOL, Tokyo, Japan) to determine the particle distribution size and morphology. The Zeta potential of NPs was determined using Dynamic Light Scattering (Zetasizer Nano-ZS, ZEN3600, Malvern instruments Ltd., Malvern, UK). Whereas, the crystalline nature of NPs was detected by X-ray diffraction.

### 2.2. Preparation of Pb and NPs Solutions and Seed Exposure

A 500 mM lead acetate (Sigma grade) stock solution was freshly prepared by adding lead acetate to deionized water and allowed to dissolve by slowly rotating the flask. Working solutions of different concentrations (0, 2, 4, 6, 8, 10, 15 and 20 mM) were prepared by dilution with deionized water from stock solution. Stock solution of ZnO-NPs (1000 mg L^−1^) and Fe_3_O_4_-NPs (1000 mg L^−1^) were freshly prepared by dispersing NPs in deionized water for 30 min using ultrasonic vibration (100 W, 40 kHz). Dilution of the stock solution with deionized water yielded NPs solutions of 0, 50, 100, 200, 300 and 500 mg L^−1^.

*B. alba* seeds were surface sterilized in 0.5% sodium hypochlorite solution (NaOCl) followed by thorough washing with deionized water. For priming, seeds were soaked in different concentrations of both the NPs for 16 h at 25 °C in incubator followed by air drying of seeds.

### 2.3. Germination Assay and Seedling Growth Measurement

For imposing lead stress, germination papers were pre-moistened with different lead acetate solution (0, 2, 4, 6, 8, 10, 15, 20 mM). A total of one hundred nano-primed and unprimed (control) seeds, each in four replicates were kept on pre-moistened (Pb solution) germination paper for germination at 25 °C in germinator. After twenty days, the percent germination of the seed was determined using normal seedlings and transformed into arcsine values for statistical analysis [30]. Ten seedlings were taken at random from each replicate for measurement of root and shoot length, and seedling dry weight by placing seedlings in oven for 48 h at 65 °C. Seedling vigour indices were calculated based on methods given by Abdul-Baki and Anderson [31].

Vigour index I=Germination %×Seedling length (cm)Vigour index II=Germination %×Seedling dry weight (mg per seedling)

Simultaneously, the growth tolerance index (%) of the seedling growth was determined by using the following formula as per Wilkins [32].

$$\mathrm{Growth}\;\mathrm{Tolerance}\;\mathrm{Index}\;\left(\%\right)=\left\{\frac{\mathrm{Growth}\;\mathrm{in}\;\mathrm{solution}\;\mathrm{with}\;\mathrm{metal}}{\mathrm{Growth}\;\mathrm{in}\;\mathrm{solution}\;\mathrm{without}\;\mathrm{metal}}\right\}\times 100$$

The remaining seedlings from different treatments were kept at −80 °C for analysis of various physio-biochemical parameters.

### 2.4. Root Growth Analysis

Root parameters such as root length density (RLD), volume, surface area, diameter and number of tips and forks were measured by using an acquired TIFF-format grey image (400 dpi resolution) with a flatbed scanner (Epson Expression 11000XL, Epson, Suwa, Japan) and the software Win-RHIZO-2013 (Regent Instrument Inc., Quebec, QC, Canada).

### 2.5. Estimation of Hydrogen Peroxide, Malondialdehyde (MDA) and Proline

The hydrogen peroxide (H_2_O_2_) content was determined according to Mukherjee and Choudhuri [33]. One gram of *B. alba* seedling was ground in 10 mL of chilled acetone and filtered using Whatman No. 1 filter paper, then 4 mL of titanium reagent (titanium dioxide and potassium sulphate digested with concentrated H_2_SO_4_) and 5 mL of ammonium solution were added to precipitate the titanium-hydro-peroxide complex. Reaction mixture was centrifuged at 10,000 rpm for 10 min. The precipitate was re-centrifuged after being dissolved in 10 mL of 2 M H_2_SO_4_. The absorbance of supernatant was measured at 415 nm and the concentration was determined using standard curve.

Malondialdehyde (MDA) content in seedlings was determined by using the method described by Heath and Parker [34]. In an ice bath, seedlings (0.2 g) were homogenized with 2 mL of 5% (*w*/*v*) trichloroacetic acid (TCA), and the extract was centrifuged at 10,000 rpm for 10 min. The supernatant was mixed with 2 mL of 0.67% (*w*/*v*) thiobarbituric acid (TBA), then incubated in a boiling water bath for 25–30 min. The supernatant was collected, and absorbance was measured at 450, 532, and 600 nm.

The proline content was calculated based on reaction of proline with ninhydrin as described by Bates et al. [35], with minor modifications. Seedling (100 mg) was homogenized in 0.1 M sulphosalicylic acid and centrifuged for 15 min at 10,000 rpm. About 2 mL of the collected supernatant was mixed with 2 mL of glacial acetic acid and 2 mL of 2.5% ninhydrin reagent before incubating for one hour in water bath at 100 °C. The reaction was stopped on ice and finally, 4 mL toluene was added to the reaction mixture and mixed properly by vortexing. Absorbance was measured at 520 nm using toluene as blank and proline content was measured by using a standard curve.

### 2.6. Assay of Antioxidant Enzyme Activities

An enzyme extract for superoxide dismutase (SOD) and catalase (CAT) was prepared by homogenizing 1 g of 20-day-old seedlings in 6.0 mL of extraction buffer [0.1 M phosphate buffer (pH 7.0) containing 0.5 mM EDTA]. The homogenate was centrifuged at 10,000 rpm for 20 min, and the resulting supernatant was used as the enzyme extract. Total protein content of extract was determined using bovine serum albumin as a standard [36].

The activity of superoxide dismutase (SOD; EC 1.15.1.1) was assayed on the basis of its ability to inhibit photochemical reduction in nitro-blue tetrazolium (NBT) resulting in the reduction in optical density at wavelength 560 nm due to formazone formed by superoxide radical and NBT dye in the presence of the enzyme [37].

Catalase (CAT; EC 1.11. 1.6) converts H_2_O_2_ into H_2_O and oxygen and its activity was measured using the method described by Aebi [38]. The enzyme extract was added to a 3 mL reaction mixture containing 50 mM potassium phosphate buffer (pH 7.0) and 10 mM hydrogen peroxide. The enzyme activity was measured by observing the reduction in absorbance at 240 nm.

The peroxidase (POD; EC 1.11.1.7) activity was measured using the protocol of Zhang et al. [39]. Oxidase activity of peroxidase is the reduction of oxygen molecule (O_2_) to superoxide (O_2_^−^) and hydrogen peroxide (H_2_O_2_). By adding 2.86 mL distilled water, a three mL reaction mixture containing potassium phosphate buffer (pH 7.0), 30% H_2_O_2_ 24 µL (2 mM), guaiacol 96 µL (16 mM), 20 µL of enzyme extract was made. Change in absorbance of reaction solution (formation of tetra-guaiacol) during every 30 s was determined at 470 nm. The extinction coefficient (∈ = 26.6 mM^−1^ cm^−1^) of oxidation products (tetra-guaiacol) was used to calculate the enzyme activity, which was expressed as µmol tetra-guaiacol formed.min^−1^ g^−1^ FW.

### 2.7. Estimation of Lead Concentration

To determine the lead (Pb) content in seedlings, 0.3 g dried samples were digested in HNO_3_ and HCl mixture (3:7 ratio), then heated on a hot plate at 70 °C until the colour turned from brown to light yellow. Following digestion, the samples were filtered with Whatman No.1 filter paper and transferred to 25 mL tubes, which were then make up to a volume of 20 mL with double distilled water [40]. Atomic Absorption Spectrometry (AAS) was used to determine the Pb content (Perkin Elmer, AAnalyst 300, Massachusetts, US). The Pb concentration in seedling was estimated following the procedures described by Monni et al. [41].

### 2.8. Computation of NPs Superiority in Alleviating Pb Stress

The superiority of NPs in alleviating the Pb stress was calculated using a nonlinear scoring method, followed by calculation of an index value [42,43]. In our study parameters namely Pb content, H_2_O_2_ and MDA was considered as a “less is better, attribute whereas the rest of the parameters were deemed ‘more is better’. The higher index score indicates which NPs was better in alleviating the Pb stress at a specified concentration. The non-linear scoring was calculated using the following formula.

(1) $$NL\left(x\right)=a/(1+{\left(x/x_{o}\right)}^{b})$$

where *NL*(*x*) is the non-linear score of the variable between 0 and 1, ‘*a*’ is the maximum value reached by the function, in our case, *a* = 1, *x* is the unknown of the equation, corresponding to the value of the parameter in question in each case, $x_{o}$ is the mean value of each parameter of the study. *b* is the value of the slope of the equation. Using *b* = −7.5, for positive function and +7.5 for negative function we obtained curve that fit in a sigmoidal tending to 1 for the parameters in study.

To calculate the NPs Performance Index (*NPI*) value, the obtained *NL*(*x*) score was multiplied by a weighted factor and value of all the parameters were added together. The weighted factor of each parameter under consideration was calculated using the principal component analysis. Weighted factor for these PC were calculated by dividing the individual PC score with the cumulative total of the all PCs.

(2) $$NPI=\overset{n}{\underset{i=1}{\sum }}\left(Wi\times Si\right)$$

where, *Si* is the score of the variable and *Wi* is the weighing factor obtained from PCA.

### 2.9. Statistical Analysis

Analysis of variance (ANOVA) was performed between treatment samples in a completely randomized design using SAS software (Version 9.3; SAS Institute, Cary, NC, USA) and the significant levels of difference between means were determined using the Duncan’s Multiple Range Test at 5% and 1% probability level. Data represented in the tables and figures are mean ± standard error (SE) of four independent replicates of each treatment.

## 3. Results

### 3.1. Characterization of ZnO- and Fe_3_O_4_-NPs

**Figure 1:**
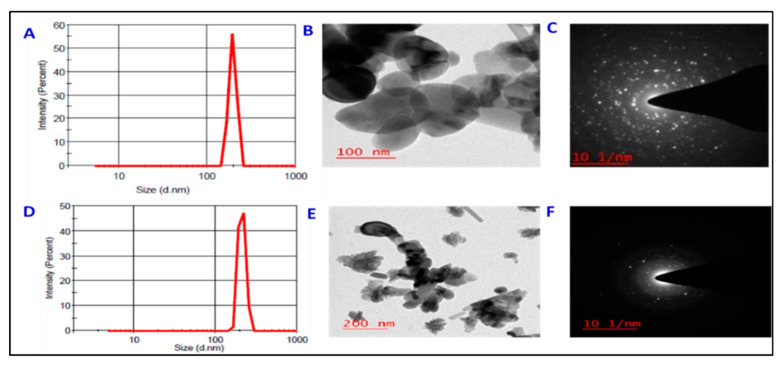
(**A**) DLS size distribution of ZnO-NPs, (**B**) TEM image of ZnO-NPs, (**C**) SAED pattern of the ZnO-nanocrystals, (**D**) DLS size distribution of Fe_3_O_4_-NPs, (**E**) TEM image of Fe_3_O_4_-NPs and (**F**) SAED pattern of the Fe_3_O_4_-nanocrystals.

Dynamic light scattering (DLS) measurement showed that, synthesized highly pure ZnO- and Fe_3_O_4_-NPs had average hydrodynamic diameter of ~193 nm and ~210 nm, respectively, which were positively correlated with their primary sizes (Figure 1). The surface area of ZnO-NPs and Fe_3_O_4_-NPs was around 20 m^2^ g^−1^ and 25 m^2^ g^−1^, respectively. The transmission electron microscopy (TEM) images revealed that ZnO-NPs were spherical with average size of ~70 nm, whereas Fe_3_O_4_-NPs were with an average primary size of ~55 nm and are quasi-spherical in shape (Figure 1B,E). The typical selected area of electron diffraction pattern (SAED) in planes (1 0 1), (1 0 2), (1 1 0), (0 0 2), (1 0 0), (1 0 3) of the ZnO-NPs with eleven sharp and bright concentric rings, confirm hexagonal structure and crystalline nature. However, selected area of electron diffraction pattern (Figure 1C,F) consists diffraction spots/rings that were indexed with correspondence to the magnetite spinal structure (4 4 0), (5 1 1), (4 2 2), (4 0 0), (3 1 1) and (2 2 0) planes which is characteristics of diffraction ring pattern of polycrystalline face centered cubic (FCC) crystal structure of Fe_3_O_4_.

### 3.2. Preliminary Study with Pb and NPs Treatments

A preliminary study was conducted to determine the phytotoxic concentration of Pb solution on *B. alba* seed germination and seedling growth using standard germination test. A dose-dependent negative relationship was established with increasing Pb concentration from 0 to 20 mM for both the parameters (Supplementary Table S1). As the Pb concentration increased from 0 to 20 mM, the seed germination, root length, shoot length, seedling biomass, vigour index-I and vigour index-II decreased by 72.8%, 92.0%, 69.1%, 91.0%, 95.1 and 97.6%, respectively. Growth inhibition at 20 mM Pb exposure was observed in the order of following parameters: root length > seedling biomass > germination > shoot length. Among all the parameters, the reduction in root length density (RLD) was 83.7% due to reduced root length and number of secondary roots. The reduction in SDW, on the other hand was primarily due to decreased germination and seedling length. At 10 and 8 mM Pb concentration, 50% inhibition over control was observed in seed germination and root length, respectively.

Another preliminary study was carried out to investigate the effect of seed priming with Zn- and Fe-NPs at various concentrations (0, 50, 100, 200 300 and 500 mg L^−1^) on seed germination and seedling growth. The growth tolerance index (GTI) revealed that seed priming with 200 mg L^−1^ concentration of the NPs resulted in the highest gains in seed germination and seedling growth parameters. The seed priming with Zn- and Fe-NPs at a concentration of 200 mg L^−1^ improved seed germination (15.9% and 18.7%), root length (14.9% and 20.9%), shoot length (19.4% and 28.7%), seedling dry weight (13.9% and 17.0%), vigour index-I (35.4% and 47.6%) and vigour index-II (31.3% and 38.1%) over the control, respectively (Supplementary Table S2). Among all the priming treatments, increase in germination and seedling growth was observed in the following order: FeNPs > ZnNPs > FeSO_4_ > ZnSO_4_ > ddH_2_O (hydro-priming) > control (without NPs). However, seed priming with both the NPs at 300 mg L^−1^ started showing negative effect, whereas NPs at 500 mg L^−1^ had a toxic effect on seed germination and seedling growth (Supplementary Table S2), as it decreased seed germination (5.2% and 3.2%), root length (19.7% and 31.4%), shoot length (25.0% and 28.4%), seedling dry weight (19.7% and 24.6%), vigour index-I (26.1% and 32.4%) and vigour index-II (24.3% and 27.4%) over the control, respectively. Additionally, results on seed quality parameters from both the NPs @ 200 mg L^−1^ were also found superior over the treatments with similar concentration of ZnSO_4_ and FeSO_4_ (Supplementary Table S2). Based on these results, a concentration of 200 mg L^−1^ was found to be optimal for studying the effect of Zn- and Fe-NPs under Pb stress conditions.

### 3.3. Effect of NPs Seed Priming under Lead Stressed Conditions

In order to observe the effect of Zn-and Fe-NPs in *B. alba* seedlings under Pb induced stress up to 20 days, various physiochemical parameters such as seed germination, seedling growth and biomass, level of Pb accumulation, photosynthetic pigment contents, ROS activity, MDA content and antioxidative enzymes activity were investigated. Following preliminary studies, five Pb concentrations (0, 4, 8, 15 and 20 mM) and two concentration (0 and 200 mg L^−1^) each for Zn- and Fe-NPs were chosen for study.

### 3.4. Effect of NPs Priming on Seed Germination and Seedling Growth under Lead Stress

The results of the effect of Pb (0, 4, 8, 15, 20 mM) in combination with Zn- and Fe-NPs (0, 200 mg L^−1^) pertaining to seed germination attributes of *B. alba* are depicted in Table 1. Interestingly, under the different concentration of Pb, the seed primed with Zn- and Fe-NPs at 200 mg L^−1^ showed significant increase in seed germination and seedling vigour. Seed primed with Zn- and Fe-NPs when exposed to 20 mM Pb showed highest recovery in seed germination (34.7% and 54.9%), root length (152.9% and 252.9%), shoot length (31.2% and 46.2%), seedling biomass (32.4% and 109.3%), vigour index-I (118.1% and 207.3%) and vigour index-II (78.4% and 224.3%), respectively. In addition, supplementation of both the NPs under Pb condition was found to increase the growth tolerance index when compared with control (Table 1).

### 3.5. Effect of Pb and NPs on Root Growth Parameters

**Figure 2:**
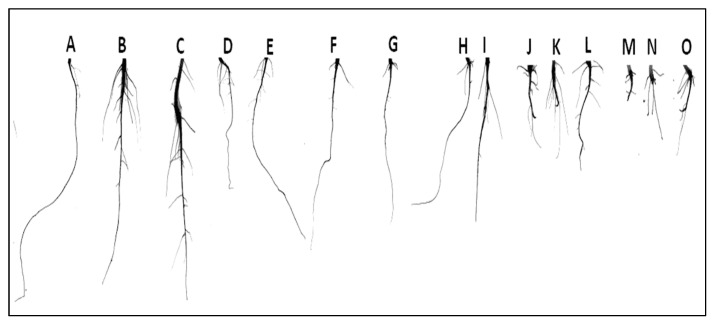
Root scanning images of 20-day-old *B. alba* seedling under different treatments, (**A**) control (0 mM Pb + 0 mg/L NPs), (**B**) ZnNPs, (**C**) FeNPs, (**D**) 4 mM Pb, (**E**) 4 mM Pb + ZnNPs, (**F**) 4 mM Pb + FeNPs, (**G**) 8 mM Pb, (**H**) 8 mM Pb + ZnNPs, (**I**) 8 mM Pb + FeNPs, (**J**) 15 mM Pb, (**K**) 15 mM Pb + ZnNPs, (**L**) 15 mM Pb + FeNPs, (**M**) 20 mM Pb, (**N**) 20 mM Pb + ZnNPs, (**O**) 20 mM Pb + FeNPs.

**Figure 3:**
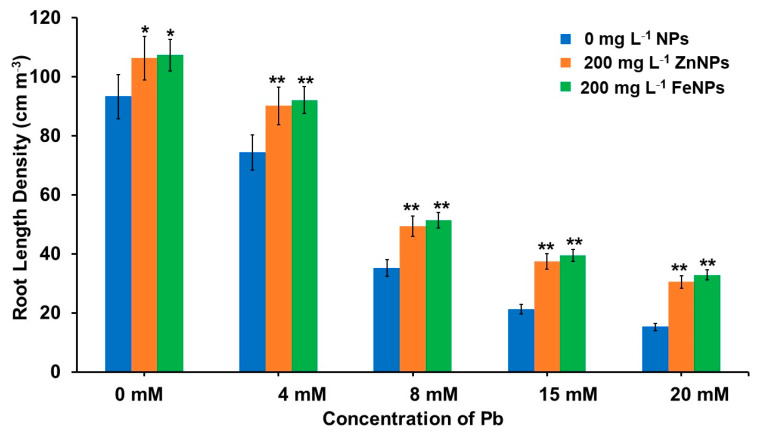
Effects of Pb and nanoparticles (ZnNPs and FeNPs) on root length density of 20-day-old *B. alba* seedling. Error bars representing the standard error. Asterisks above the error bars denote significant differences between treatments in comparison to respective treatment without NPs (* = *p* value < 0.05 and ** = *p* value < 0.01, Duncan’s Multiple Range Test).

The phenotypic differences of *B. alba* roots in root scanning (Figure 2) showed that under increasing Pb stress (0 to 20 mM) the root length and root length density (RLD), decreased by 91.97% and 83.68%, respectively (Figure 3). However, maximum RLD of Zn- and Fe-NPs primed seedlings increased 100.46% and 116.11%, respectively, under 20 mM Pb stress condition. Under the Pb stress, the seeds primed with NPs exhibited enhanced root growth by increasing secondary roots and RLD.

### 3.6. Effect of NPs Priming on Pb Content under Pb Stress Condition

**Figure 4:**
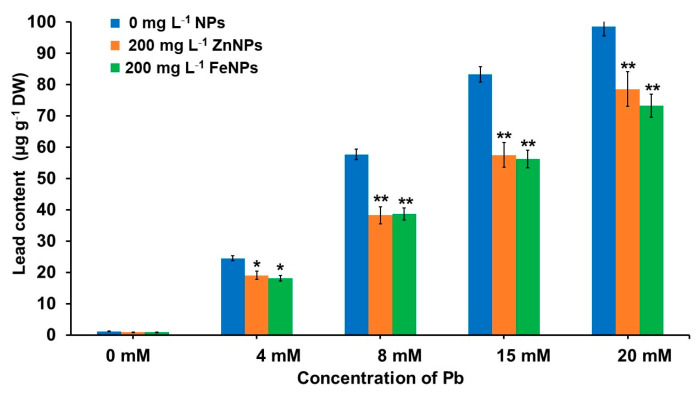
Effects of Pb and nanoparticles (ZnNPs and FeNPs) on lead content of 20-day-old *B. alba* seedling. Error bars representing the standard error. Asterisks above the error bars denote significant differences between treatments in comparison to respective treatment without NPs (* = *p* value < 0.05 and ** = *p* value < 0.01, Duncan’s Multiple Range Test).

Accumulation of Pb in NPs primed *B. alba* seedling under different Pb stress conditions is depicted in Figure 4. The accumulation of Pb content in seedlings increased from 1.14 µg g^−1^ DW at 0 mM to 98.51 µg g^−1^ DW at 20 mM. It is noteworthy to mention that the accumulation of Pb content in seedling decreased significantly in the different Pb + ZnNPs and Pb + FeNPs treatments, to 20.2–33.7% and 24.6–32.6% respectively.

### 3.7. Effect of NPs Priming on H_2_O_2_, MDA and Proline under Pb Stress

**Figure 5:**
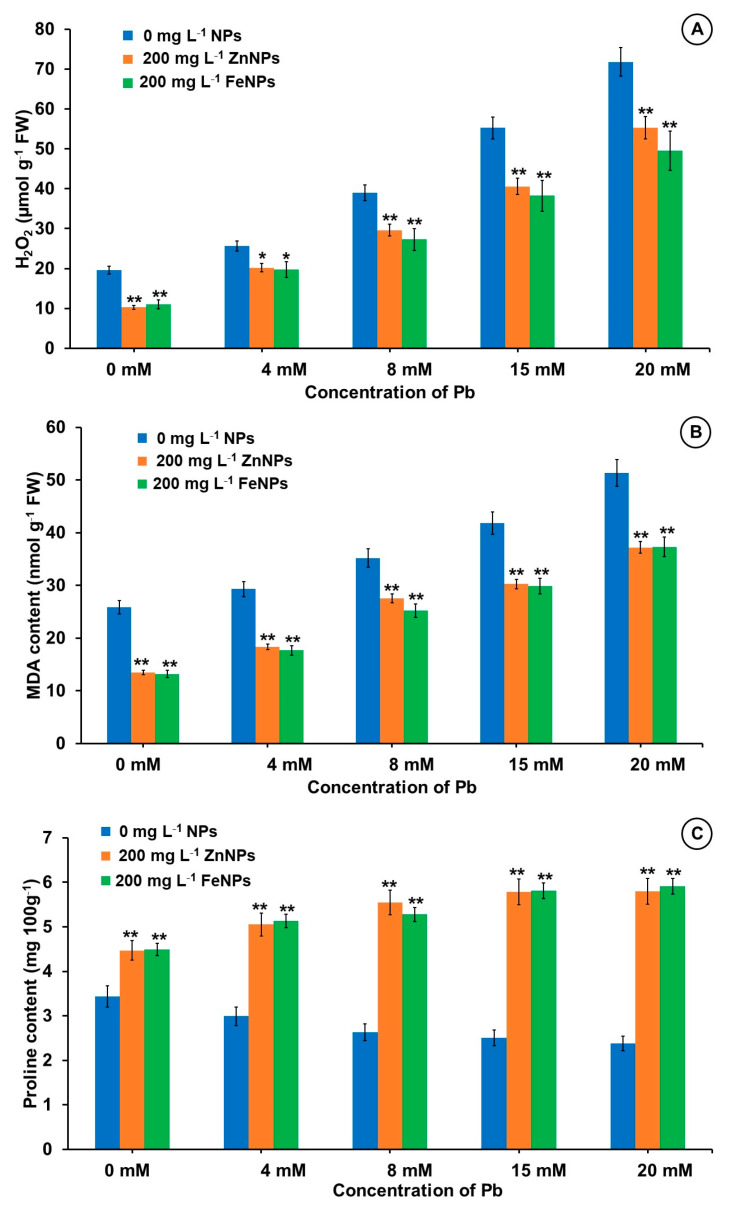
Effects of Pb and nanoparticles (ZnNPs and FeNPs) on (**A**) H_2_O_2_ content, (**B**) MDA content and (**C**) proline content of 20-day-old *B. alba* seedling. Error bars representing the standard error. Asterisks above the error bars denote significant differences between treatments in comparison to respective treatment without NPs (* = *p* value < 0.05 and ** = *p* value < 0.01, Duncan’s Multiple Range Test).

To elucidate the effect of seed priming with NPs under Pb treatments oxidative stress induced lipid peroxidation (MDA content), H_2_O_2_ content and proline content in seedlings was measured. Increasing the concentration of Pb from 0 to 20 mM resulted in significant increase in H_2_O_2_ content by 267.2% over the control (Figure 5). However, the H_2_O_2_ content of ZnNPs and FeNPs primed seedlings was significantly reduced in different Pb stressed conditions, maximum decrease 47.5% and 41.6%, respectively was observed under 20 mM Pb stress.

The MDA content in Pb treatments increased up to 98.5% at 20 mM Pb treatment as compared to Pb less control, whereas, at same Pb concentration it decreased to 27.5% and 27.3% with ZnNPs and FeNPs priming, respectively (Figure 5).

The proline content of Pb-treated seedlings decreased by 30.6% in 20 mM Pb concentration when compared to control (0 mM Pb), while in primed seedlings (Zn- and Fe-NPs) treated with various doses of Pb, the proline content increased significantly. Maximum increase in proline content 143.7% with ZnNPs and 148.3% with FeNPs was recorded in 20 mM Pb treated seedlings over NPs less respective treatment (Figure 5).

### 3.8. Effect of NPs Priming on Antioxidant Enzymes under Pb Stress

**Figure 6:**
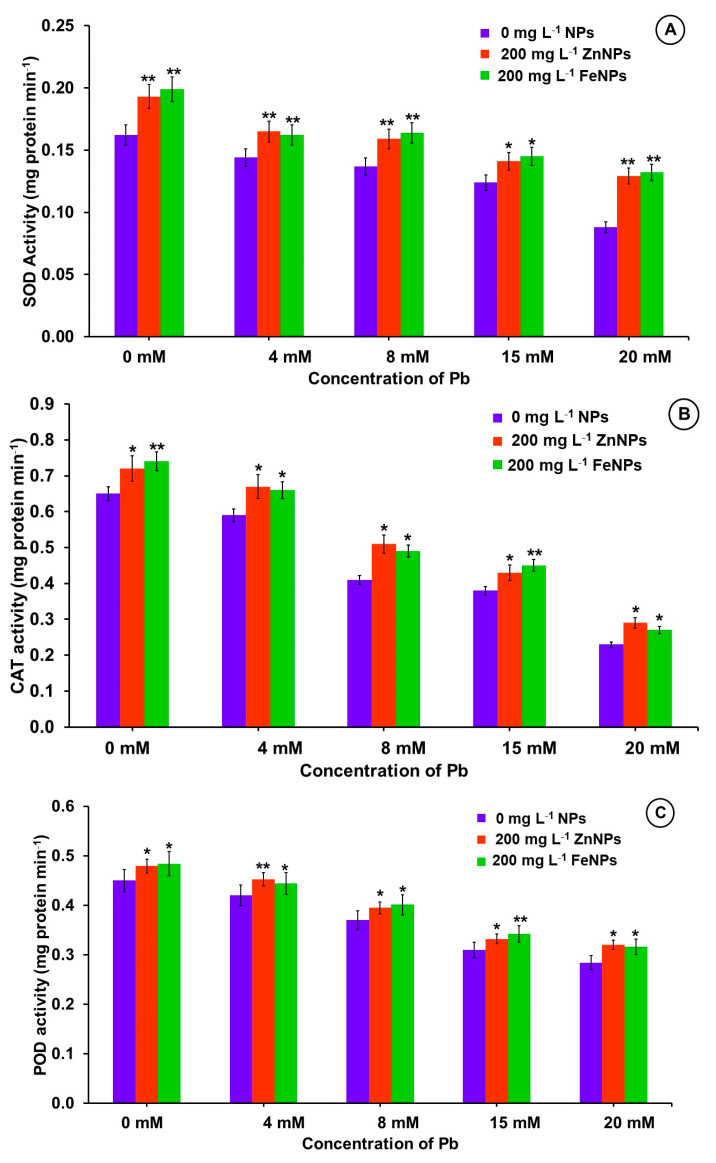
Effects of Pb and nanoparticles (Zn- and Fe-NPs) on (**A**) SOD, (**B**) CAT and (**C**) POD activity of 20-day-old *B. alba* seedling. Error bars representing the standard error. Asterisks above the error bars denote significant differences between treatments in comparison to respective treatment without NPs (* = *p* value < 0.05 and ** = *p* value < 0.01, Duncan’s Multiple Range Test).

Results showed that increasing Pb concentration in growing media from 0 to 20 mM significantly reduced the activity of SOD, CAT and POD in *B. alba* seedlings by 45.7%, 64.6% and 36.9%, respectively, compared to unstressed treatment (Figure 6). In contrast, seed priming with Zn and Fe-NPs alone increased SOD activity by 19.1% and 22.8%, CAT activity by 10.8% and 13.8%, and POD activity by 6.4% and 7.6% respectively, over the unprimed treatment.

On the other hand, treatment combinations of ZnNPs + Pb and FeNPs + Pb significantly increased SOD, CAT and POD activity over the unprimed and Pb-free treatments (Figure 6). Seed priming with NPs showed maximum percent increase in SOD activity 50.0% with FeNPs, 26.1% CAT activity with ZnNPs and 19.5% POD activity with ZnNPs was recorded in 20 mM Pb treated seedlings over the unprimed treatment (Figure 6).

NPI score estimated for the studied parameters indicated that FeNPs has a high NPI score under various Pb treatments over ZnNPs, indicating the superiority in alleviating Pb stress in the treated seedlings. In case of 20 mM Pb + 200 mg L^−1^ ZnNPs treatment the obtained score was 0.110 and the score of the 20 mM Pb + 200 mg L^−1^ FeNPs treatment was 0.116, indicating the superiority of FeNPs in alleviating Pb stress (Table 1). The *t*-test of means revealed a significant difference between the Fe- and Zn- NPs score treatments means.

## 4. Discussion

### 4.1. Effect of Lead and NPs Priming on Germination and Seedling Growth

Pb serves no biological function in the plant system and is classified as a toxic pollutant because of its unintended effects on morphological, physiological, and biochemical functions of plants [7]. During seed germination in contaminated media, the seed coat, first and foremost prevents Pb from entering into the seed until it is ruptured by the developing radicle. Pb enters the seed after seed coat ruptures and hampers the seed germination by impairing radicle and hypocotyl growth eventually affecting the root system [11]. Pb ions uptake through roots is a nonselective H^+^/ATPases driven process, which is further transported by apoplastic movement, consequently metal ion deposition in the endodermis and finally transported by symplastic movement [44]. This study showed that Pb treated seedlings had blackened roots, decreased RLD, lower root length and diameter and fewer lateral or secondary roots. Germination and radicle elongation are found to be more sensitive to Pb toxicity because of the first exposure [45,46,47]. The accumulation of Pb in the root meristem leads to disruption of micro-fibrils and micro-tubules, activation of certain wall-degrading enzymes and disbalance in mineral homeostasis [48]. At higher concentrations (15 mM and 20 mM) of Pb exposure, germination and root growth was almost checked, which could be due to decreased water uptake ability, impaired reserve mobilization by interfering the enzymes such as amylase and proteases, altered transpiration strength and consequently inhibition of cell division in the root tip or cell death [49].

Tolerating abiotic stress by establishing seedlings more uniformly requires a robust seed with enhanced seed vigour, which include rapid germination, early radicle emergence, rapid shoot and root growth, and long mesocotyls and coleoptiles [50]. Among the various seed vigour enhancement treatments, nano priming is relatively new and efficient as required at very low concentrations and does not involve soil application, thereby preventing the dispersal of large amounts of NPs into the ecosystems [51]. Previous studies have shown that NPs application has a dose-dependent response on seed germination and seedling growth by altering the physio-biochemical response such as water absorption activity, enzymatic activity, expression of specific mRNA, antioxidants activity and starch metabolism in various crops i.e., *Zea mays*, *Triticum aestivum*, *Oryza sativa* and *Lactuca sativa* [52,53,54,55,56]. Moreover, the role of seed priming through various agents in Pb stress mitigation is also reported [57,58,59], although the NPs seed priming for Pb stress alleviation in not exploited. This study shows that priming of *B. alba* seeds with Zn- and Fe-NPs at 200 mg L^−1^ concentration for 16 h at 25 °C improves germination and seedling vigour, whereas the seed priming at 500 mg L^−1^ with both the NPs reduced germination, root growth, and seedling growth.

This finding showed that seed priming with Zn- and Fe-NPs at 200 mg L^−1^ alleviated Pb toxicity on seed germination, root development, shoot growth seedling biomass and seed vigour under different Pb stress conditions. Increased root growth after the application of NPs was because of increased root length and diameter, RLD, number of secondary roots and root tips. These priming treatments alleviate Pb stress in the following order: FeNPs > ZnNPs > hydropriming > control (untreated).

### 4.2. Effect of NPs Priming on Pb Uptake

A decrease in Pb content in seedling was observed in Zn- and Fe-NPs primed seeds under Pb stressed conditions, which may contribute in mitigation of Pb stress during seed germination and root growth. The possible mechanism of Pb alleviation by NPs application may be as follows (i) increased synthesis and deposition of callose as a barrier to stops Pb entry [60] (ii) sequestration of heavy metal in the vacuole [60,61] (iii) precipitation of NPs on root surface to prevents Pb entry [62] (iv) translocation of Pb to the above ground parts of hyperaccumulator plants such as *Brassica juncea* [63] (v) formation of phyto-chelatins and proline-heavy metal complex [64] (vi) upregulation of heavy metal tolerance related genes such as HMA4 in *Arabidopsis helleri* [65] (vii) transportation of cations through common transporter; small size favors the transport of NPs [66,67]. Pb and other heavy metals are transported through the common metal transporters such as Zrt (zinc-regulated transporters) IRT-like proteins (ZIP) and the cation efflux family. The small size of Zn- and Fe-NPs provides advantage in uptake and translocation over the larger size of Pb. Similarly, the treatment of wheat seeds with iron, zinc, and manganese nanoparticles resulted in higher contents of respective minerals in seeds and plants [68].

### 4.3. Pb Induced Physio-Biochemical Changes and Its Mitigation by NPs

Multiple physiological and biochemical mechanisms are expected to be involved in Zn- and Fe-NPs promoted germination and seedling growth under Pb stress condition. We attempted to determine the adaptive mitigation strategies used by NPs treated seeds under Pb stress conditions particularly response of ROS and antioxidants (SOD, CAT, POD and Proline) during germination and early seedling growth. Normally, a balance between the generation and destruction of ROS is required for seed germination and seedling growth. Furthermore, the tiny size (<100 nm) of NPs allows for easy penetration into cells and regulates water activity, which controls seed germination and plant growth [69], whereas a larger surface area allows for more adsorption and targeted delivery of substances [20]. Under lead stress condition, plants produce an excess amount of ROS such as super oxide anions (radical O_2_^−.^), hydroxyl radicals and hydrogen peroxide either directly through Haber-Weiss reactions or indirectly through oxidative stress as primary response [44]. These ROS not only cause abnormal cell metabolism, lipid peroxidation, membrane damages and altered gene expression but also cause cell death when they react with cell membranes, organelles, proteins, lipids, and nucleotides [70]. In the current study, 15 mM and 20 mM Pb concentration causes a significant increase in H_2_O_2_ generation due to oxidative stress, consequently lipid peroxidation and an increase in MDA content. Zn- and Fe-NPs primed seedling under Pb stress, on the other hand showed reduced oxidative stress due to decrease in H_2_O_2_ content and decrease in MDA content mediated lipid peroxidation. As MDA is the major product of membrane lipid peroxidation, a higher MDA content in 20 mM Pb stressed plants indicates more membrane damage over control seedlings. Under Pb stress, similar results were observed in *Alocasia macrorrhiza* [71].

To counteract the oxidative stress caused by Pb, higher antioxidant enzyme activity (SOD, CAT and POD) was observed when seeds were primed with Zn- and Fe-NPs at 200 mg L^−1^. Plants activate certain enzymatic (CAT, APX, SOD etc.) and non-enzymatic antioxidants (proline, cysteine, non-protein thiol, ascorbic acid, glutathione etc.) as defense mechanisms to eliminate excess of ROS [72]. SOD is the primary enzymatic antioxidant that catalyses the detoxification of superoxide radicals, into H_2_O_2_ and molecular oxygen. This results in an increase in H_2_O_2_ concentration inside the cell, but other two scavenging enzymes catalase and peroxidase convert it to H_2_O and O_2_ [73].

Proline is one of the most important osmolytes and non-enzymatic antioxidant involved in scavenging ROS molecules by activating antioxidant defence mechanisms, assisting in cell osmotic balance adjustment, and acting as a saviour during abiotic stress by providing energy and nutrients for plants [74]. In absence of NPs, proline concentration decreased with the increase in Pb concentration from 0, 4, 8, 15 and 20 mM. Proline content increased significantly, not only in NPs primed seedling but also in NPs primed + Pb stressed treatments. The synergistic effect of increased proline content and antioxidants in seedling under Pb stress conditions may explain the positive effect of NPs seed priming on reduced oxidative stress. This helps in the maintenance of the cell’s redox status by reducing H_2_O_2_ and MDA levels. Proline also functions as a heavy metal chelator, there by alleviating heavy metal stress by formation of phytochelatins and proline-heavy metal complexes [64].

In our study, an increase in H_2_O_2_, MDA, and proline content as well as a decrease in antioxidant activity such as SOD, CAT and POD, clearly demonstrated increased oxidative stress and cytotoxicity in the presence of Pb. Zn- and Fe-NPs seed priming, however, mitigated oxidative stress by increasing antioxidant activity (SOD, CAT and POD), proline content, and decreasing H_2_O_2_ and MDA content. The relationship between the decrease in MDA and H_2_O_2_ content and the increase in SOD and catalase activities in the presence of Zn- and Fe-NPs suggests that the decrease in MDA in *B. alba* seedlings under Pb stress may be due to increased antioxidant activity and decreased ROS activity. Increased antioxidants activity may alleviate Pb stress through NPs by upregulating antioxidant proteins such as, SOUL heme-binding family protein (F4K452), glutathione reductase (P48641, P42770, GHR), linoleate 9S-lipoxygenase 1 (Q06327) and lipoxygenase (P38418, Q9FNX8, LPX) [75]. The high SOD activity in NPs primed seed could be attributed to increased binding of Zn^2+^ to thiols [76], which stimulated SOD synthesis [77].

## 5. Conclusions

The presence of Pb in growth medium is a serious issue, causing phytotoxicity on seed germination, root growth, and shoot growth of *B. alba* due to the accumulation of Pb, excessive generation of ROS leading to oxidative stress, lipid peroxidation, and suppression of antioxidant activities. The present study demonstrates that seed priming with ZnNPs and FeNPs at 200 mg L^−1^ has the potential to alleviate Pb induced damage in *B. alba*, through activating a plant’s defense mechanism. Under Pb stress conditions, seed priming with the NPs increased seed germination, seedling growth, particularly root growth and RLD, and seed vigour by reducing Pb uptake, H_2_O_2_ activity, and MDA content and increased proline content and antioxidant enzymes (SOD, CAT and POD) activities. As a result, seed priming with either Zn- and Fe-NPs may be a better strategy for alleviating the Pb stress, particularly during early seedling growth. The future thrust will be toward the study of the synergistic effect of both of the NPs in seed germination and seedling growth.

## Figures and Tables

**Table 1:** Effects of Pb treatment along with nanoparticles (ZnNPs and FeNPs @ 200 mg L^−1^) on seed germination, seedlings growth, seedling biomass, vigour indices and Growth Tolerance Index (GTI) in 20 days old *B. alba* seedling.

Table. *Cont.*	Germination (%)	Root Length (cm)	Shoot Length (cm)	Seedling Biomass (mg Seedling^−1^)	Vigour Index-I	Vigour Index-II	NPI Score	GTI (%)		
								Root Tissue	Shoot Tissue	Seedling Biomass
0 mM Pb	71.0 ± 0.82 ^b^	15.1 ± 0.24 ^b^	11.6 ± 0.11 ^c^	26.86 ± 0.08 ^b^	1895.0 ± 12.1 ^c^	1907.1 ± 3.7 ^b^	0.840	-	-	-
ZnNPs	82.3 ± 0.62 ^a^	17.3 ± 0.11 ^a^	13.9 ± 0.15 ^b^	30.42 ± 0.19 ^a^	2566.1 ± 8.5 ^b^	2503.6 ± 3.3 ^a^	0.931	114.9	119.4	113.3
FeNPs	84.3 ± 0.67 ^a^	18.2 ± 0.10 ^a^	15.0 ± 0.10 ^a^	31.24 ± 0.17 ^a^	2796.2 ± 10.5 ^a^	2633.5 ± 5.1 ^a^	0.936	120.9	128.7	116.3
4 mM Pb	65.0 ± 0.72 ^cd^	13.1 ± 0.18 ^c^	9.4 ± 0.09 ^e^	18.96 ± 0.11 ^c^	1461.9 ± 13.5 ^e^	1232.4 ± 2.2 ^d^	0.721	86.7	81.2	70.6
4 mM Pb + ZnNPs	68.7 ± 0.87 ^bc^	14.7 ± 0.15 ^b^	10.0 ± 0.07 ^d^	19.40 ± 0.16 ^c^	1696.9 ± 9.7 ^d^	1332.8 ± 6.1 ^cd^	0.879	97.5	86.1	72.2
4 mM Pb + FeNPs	71.0 ± 0.59 ^b^	15.7 ± 0.16 ^b^	11.8 ± 0.10 ^c^	20.69 ± 0.14 ^c^	1951.1 ± 11.3 ^c^	1469.0 ± 2.8 ^c^	0.900	104.2	101.4	77.0
8 mM Pb	49.3 ± 0.61 ^e^	8.1 ± 0.20 ^e^	7.9 ± 0.13 ^fg^	11.65 ± 0.09 ^e^	786.3 ± 14.5 ^g^	574.3 ± 1.8 ^f^	0.192	53.7	67.6	43.4
8 mM Pb + ZnNPs	62.7 ± 0.45 ^d^	8.9 ± 0.17 ^e^	8.8 ± 0.08 ^ef^	12.63 ± 0.21 ^de^	1106.0 ± 9.9 ^f^	791.9 ± 4.3 ^e^	0.524	59.0	75.3	47.0
8 mM Pb + FeNPs	66.3 ± 0.71 ^bcd^	11.2 ± 0.32 ^d^	9.9 ± 0.07 ^d^	13.64 ± 0.07 ^d^	1398.3 ± 10.1 ^e^	904.3 ± 3.8 ^e^	0.643	74.1	85.4	50.8
15 mM Pb	31.3 ± 0.69 ^g^	3.5 ± 0.28 ^g^	5.3 ± 0.11 ^i^	4.53 ± 0.14 ^g^	276.4 ± 12.5 ^j^	141.8 ± 2.9 ^ghi^	0.044	23.3	45.8	16.9
15 mM Pb + ZnNPs	38.0 ± 0.55 ^f^	4.0 ± 0.14 ^g^	6.5 ± 0.14 ^h^	5.37 ± 0.15 ^fg^	399.4 ± 11.9 ^i^	204.1 ± 5.7 ^gh^	0.192	26.8	55.7	20.0
15 mM Pb + FeNPs	41.0 ± 0.72 ^f^	6.5 ± 0.18 ^f^	7.7 ± 0.07 ^g^	6.51 ± 0.09 ^f^	581.8 ± 7.8 ^h^	266.9 ± 4.9 ^g^	0.235	42.9	66.4	24.2
20 mM Pb	19.3 ± 0.19 ^h^	1.2 ± 0.22 ^h^	3.6 ± 0.10 ^j^	1.82 ± 0.11 ^i^	92.6 ± 5.2 ^k^	35.1 ± 1.6 ^i^	0.010	8.0	30.9	6.8
20 mM Pb + ZnNPs	26.0 ± 0.23 ^g^	3.1 ± 0.16 ^g^	4.7 ± 0.09 ^i^	2.41 ± 0.17 ^hi^	202.0 ± 4.4 ^j^	62.7 ± 4.1 ^hi^	0.110	20.3	40.5	9.0
20 mM Pb + FeNPs	29.9 ± 0.35 ^g^	4.3 ± 0.23 ^g^	5.3 ± 0.14 ^i^	3.81 ± 0.19 ^gh^	284.6 ± 8.8 ^j^	113.9 ± 1.9 ^ghi^	0.116	28.3	45.2	14.2

Values in the table are the mean of four replications ± standard error (SE). Different letters in each row are statistically significant at *p* < 0.05 level. Germination values are expressed in percentage and transformed to the respective angular (arc sin) values before subjecting them to statistical analysis.

## Data Availability

Data are available from the authors upon request.

## Supplementary Materials

The following supporting information can be downloaded at: https://www.mdpi.com/article/10.3390/plants11172227/s1, Table S1: Effects of different Pb concentrations on seed germination, seedlings growth, seedling biomass, vigour indices and Growth Tolerance Index in 20 days old B. alba seedling; Table S2: Effects of seed priming with NPs and non-NPs on seed germination, seedlings growth, seedling biomass, vigour indices and Growth Tolerance Index (GTI) in 20 days old B. alba seedling.

## References

[1] Costa R.G., Araújo C.F.d.S., Ferreol Bah A.H., Junior E.A.G., Rodrigues Y.J.d.M., Menezes-Filho J.A. (2018). Lead in mangrove root crab (*Goniopsis Cruentata*) and risk assessment due to exposure for estuarine villagers. Food Addit. Contam. Part B.

[2] Debnath B., Singh W., Manna K. (2019). Sources and toxicological effects of lead on human health. Indian J. Med. Spec..

[3] Pourrut B., Shahid M., Dumat C., Winterton P., Pinelli E. (2011). Lead uptake, toxicity, and detoxification in plants. Reviews of Environmental Contamination and Toxicology.

[4] Flora G., Gupta D., Tiwari A. (2012). Toxicity of lead: A review with recent updates. Interdiscip. Toxicol..

[5] Fu Z., Xi S. (2020). The effects of heavy metals on human metabolism. Toxicol. Mech. Methods.

[6] Kumar A., Kumar A., M.M.S. C.-P., Chaturvedi A.K., Shabnam A.A., Subrahmanyam G., Mondal R., Gupta D.K., Malyan S.K., Kumar S.S. (2020). Lead toxicity: Health hazards, influence on food chain, and sustainable remediation approaches. Int. J. Environ. Res. Public Health.

[7] Zulfiqar U., Farooq M., Hussain S., Maqsood M., Hussain M., Ishfaq M., Ahmad M., Anjum M.Z. (2019). Lead toxicity in plants: Impacts and remediation. J. Environ. Manag..

[8] Purushotam D., Lone M.A., Rashid M., Rao A.N., Ahmed S. (2012). Deciphering heavy metal contamination zones in soils of a granitic terrain of Southern India using factor analysis and GIS. J. Earth Syst. Sci..

[9] Hadi F., Aziz T. (2015). A mini review on lead (Pb) toxicity in plants. J. Biol. Life Sci..

[10] Aslam M., Aslam A., Sheraz M., Ali B., Ulhassan Z., Najeeb U., Zhou W., Gill R.A. (2021). Lead toxicity in cereals: Mechanistic insight into toxicity, mode of action, and management. Front. Plant Sci..

[11] Seneviratne M., Rajakaruna N., Rizwan M., Madawala H.M.S.P., Ok Y.S., Vithanage M. (2019). Heavy metal-induced oxidative stress on seed germination and seedling development: A critical review. Environ. Geochem. Health.

[12] Singh H.P., Kaur G., Batish D.R., Kohli R.K. (2011). Lead (Pb)-inhibited radicle emergence in *Brassica campestris* involves alterations in starch-metabolizing enzymes. Biol. Trace Elem. Res..

[13] Karalija E., Selović A., Dahija S., Demir A., Samardžić J., Vrobel O., Ćavar Zeljković S., Parić A. (2021). Use of seed priming to improve cd accumulation and tolerance in *Silene sendtneri*, novel cd hyper-accumulator. Ecotoxicol. Environ. Saf..

[14] Khan A.R., Azhar W., Wu J., Ulhassan Z., Salam A., Zaidi S.H.R., Yang S., Song G., Gan Y. (2021). Ethylene participates in zinc oxide nanoparticles induced biochemical, molecular and ultrastructural changes in rice seedlings. Ecotoxicol. Environ. Saf..

[15] Monreal C.M., DeRosa M., Mallubhotla S.C., Bindraban P.S., Dimkpa C. (2016). Nanotechnologies for increasing the crop use efficiency of fertilizer-micronutrients. Biol. Fertil. Soils.

[16] Sharma V., Kumar A., Dhawan A. (2012). Nanomaterials: Exposure, effects and toxicity assessment. Proc. Natl. Acad. Sci. USA India Sect. B Biol. Sci..

[17] Khan A.R., Wakeel A., Muhammad N., Liu B., Wu M., Liu Y., Ali I., Zaidi S.H.R., Azhar W., Song G. (2019). Involvement of ethylene signaling in zinc oxide nanoparticle-mediated biochemical changes in *Arabidopsis thaliana* leaves. Environ. Sci. Nano.

[18] Salam A., Khan A.R., Liu L., Yang S., Azhar W., Ulhassan Z., Zeeshan M., Wu J., Fan X., Gan Y. (2022). Seed priming with zinc oxide nanoparticles downplayed ultrastructural damage and improved photosynthetic apparatus in maize under cobalt stress. J. Hazard. Mater..

[19] Singh J., Lee B.-K. (2016). Influence of Nano-TiO2 particles on the bioaccumulation of cd in soybean plants (*Glycine max*): A possible mechanism for the removal of Cd from the contaminated soil. J. Environ. Manag..

[20] Ji Y., Zhou Y., Ma C., Feng Y., Hao Y., Rui Y., Wu W., Gui X., Le V.N., Han Y. (2017). Jointed toxicity of TiO2 NPs and Cd to rice seedlings: NPs alleviated Cd toxicity and Cd promoted NPs uptake. Plant Physiol. Biochem..

[21] Andreini C., Banci L., Bertini I., Rosato A. (2006). Zinc through the three domains of life. J. Proteome Res..

[22] Coleman J.E. (1998). Zinc enzymes. Curr. Opin. Chem. Biol..

[23] Chang C.Y., Yu H.Y., Chen J.J., Li F.B., Zhang H.H., Liu C.P. (2014). Accumulation of heavy metals in leaf vegetables from agricultural soils and associated potential health risks in the pearl river delta, South China. Environ. Monit. Assess..

[24] Zhou H., Yang W.-T., Zhou X., Liu L., Gu J.-F., Wang W.-L., Zou J.-L., Tian T., Peng P.-Q., Liao B.-H. (2016). Accumulation of heavy metals in vegetable species planted in contaminated soils and the health risk assessment. Int. J. Environ. Res. Public Health.

[25] Ulhassan Z., Gill R.A., Huang H., Ali S., Mwamba T.M., Ali B., Huang Q., Hamid Y., Khan A.R., Wang J. (2019). Selenium mitigates the chromium toxicity in *Brassicca napus* L. by ameliorating nutrients uptake, amino acids metabolism and antioxidant defense system. Plant Physiol. Biochem..

[26] Ulhassan Z., Gill R.A., Ali S., Mwamba T.M., Ali B., Wang J., Huang Q., Aziz R., Zhou W. (2019). Dual behavior of Selenium: Insights into physio-biochemical, anatomical and molecular analyses of four *Brassica napus* cultivars. Chemosphere.

[27] Sagar V., Pragya, Bhardwaj R., Devi J., Singh S.K., Singh P., Singh J. (2022). The inheritance of betalain pigmentation in *Basella alba* L.. S. Afr. J. Bot..

[28] Singh J., Devi J., Sagar V. (2022). Vegetable biofortification: An underexploited silver lining for malnutrition management. Biofortification of Staple Crops.

[29] Vallabani N.V.S., Karakoti A.S., Singh S. (2017). ATP-mediated Intrinsic peroxidase-like activity of Fe_3_O_4_ -based nanozyme: One step detection of blood glucose at physiological Ph. Colloids Surf. B Biointerfaces.

[30] ISTA (2015). Seed Testing Rules.

[31] Abdul-Baki A.A., Anderson J.D. (1973). Vigor determination in soybean seed by multiple criteria. Crop Sci..

[32] Wilkins D.A. (1978). The measurement of tolerance to edaphic factors by means of root growth. New Phytol..

[33] Mukherjee S.P., Choudhuri M.A. (1983). Implications of water stress-induced changes in the levels of endogenous ascorbic acid and hydrogen peroxide in vigna seedlings. Physiol. Plant..

[34] Heath R.L., Packer L. (1968). Photoperoxidation in isolated chloroplasts. Arch. Biochem. Biophys..

[35] Bates L.S., Waldren R.P., Teare I.D. (1973). Rapid determination of free proline for water-stress studies. Plant Soil.

[36] Bradford M. (1976). A rapid and sensitive method for the quantitation of microgram quantities of protein utilizing the principle of protein-dye binding. Anal. Biochem..

[37] Rico C.M., Morales M.I., McCreary R., Castillo-Michel H., Barrios A.C., Hong J., Tafoya A., Lee W.-Y., Varela-Ramirez A., Peralta-Videa J.R. (2013). Cerium oxide nanoparticles modify the antioxidative stress enzyme activities and macromolecule composition in rice seedlings. Environ. Sci. Technol..

[38] Aebi H. (1984). [13] Catalase in vitro. Methods in Enzymology.

[39] Zhang R., Zhang H., Tu C., Hu X., Li L., Luo Y., Christie P. (2015). Phytotoxicity of ZnO nanoparticles and the released Zn(II) ion to corn (*Zea mays* L.) and cucumber (*Cucumis sativus* L.) during germination. Environ. Sci. Pollut. Res..

[40] Zaier H., Ghnaya T., Lakhdar A., Baioui R., Ghabriche R., Mnasri M., Sghair S., Lutts S., Abdelly C. (2010). Comparative study of pb-phytoextraction potential in *Sesuvium portulacastrum* and *Brassica juncea*: Tolerance and accumulation. J. Hazard. Mater..

[41] Monni S., Salemaa M., Millar N. (2000). The tolerance of *Empetrum nigrum* to copper and nickel. Environ. Pollut..

[42] Bastida F., Luis Moreno J., Hernández T., García C. (2006). Microbiological degradation index of soils in a semiarid climate. Soil Biol. Biochem..

[43] Sagar V., Yadav R., Gaikwad K.B., Gupta S. (2016). Exploring indicator scoring as a selection tool in plant breeding: A study under conservation vs conventional tillage systems. Indian J. Genet. Plant Breed..

[44] Kohli S.K., Handa N., Bali S., Khanna K., Arora S., Sharma A., Bhardwaj R., de Voogt P. (2020). Current scenario of Pb toxicity in plants: Unraveling plethora of physiological responses. BT-Reviews of Environmental Contamination and Toxicology.

[45] Romdhane L., Panozzo A., Radhouane L., Dal Cortivo C., Barion G., Vamerali T. (2021). Root characteristics and metal uptake of maize (*Zea mays* L.) under extreme soil contamination. Agronomy.

[46] Ali S., Shahbaz M., Shahzad A.N., Khan H.A.A., Anees M., Haider M.S., Fatima A. (2015). Impact of copper toxicity on stone-head cabbage (*Brassica oleracea* Var. capitata) in hydroponics. PeerJ.

[47] Usman K., Abu-Dieyeh M.H., Zouari N., Al-Ghouti M.A. (2020). Lead (Pb) bioaccumulation and antioxidative responses in *Tetraena qataranse*. Sci. Rep..

[48] Cokkizgin A., Cokkizgin H. (2010). Effects of lead (PbCl2) stress on germination of lentil (*Lens culinaris* Medic.) lines. African J. Biotechnol..

[49] Huang T.-L., Huang H.-J. (2008). ROS and CDPK-like kinase-mediated activation of MAP kinase in rice roots exposed to lead. Chemosphere.

[50] Finch-Savage W.E., Bassel G.W. (2016). Seed vigour and crop establishment: Extending performance beyond adaptation. J. Exp. Bot..

[51] Mahakham W., Sarmah A.K., Maensiri S., Theerakulpisut P. (2017). Nanopriming technology for enhancing germination and starch metabolism of aged rice seeds using phytosynthesized silver nanoparticles. Sci. Rep..

[52] Xin X., Zhao F., Rho J.Y., Goodrich S.L., Sumerlin B.S., He Z. (2020). Use of polymeric nanoparticles to improve seed germination and plant growth under copper stress. Sci. Total Environ..

[53] Tondey M., Kalia A., Singh A., Dheri G.S., Taggar M.S., Nepovimova E., Krejcar O., Kuca K. (2021). Seed priming and coating by nano-scale zinc oxide particles improved vegetative growth, yield and quality of fodder maize (*Zea mays*). Agronomy.

[54] Sharifan H., Moore J., Ma X. (2020). Zinc oxide (ZnO) nanoparticles elevated iron and copper contents and mitigated the bioavailability of lead and cadmium in different leafy greens. Ecotoxicol. Environ. Saf..

[55] Dimkpa C.O., McLean J.E., Latta D.E., Manangón E., Britt D.W., Johnson W.P., Boyanov M.I., Anderson A.J. (2012). CuO and ZnO nanoparticles: Phytotoxicity, metal speciation, and induction of oxidative stress in sand-grown wheat. J. Nanopartic. Res..

[56] Boonyanitipong P., Kositsup B., Kumar P., Baruah S., Dutta J. (2011). Toxicity of ZnO and TiO_2_ nanoparticles on germinating rice seed *Oryza sativa* L.. Int. J. Biosci. Biochem. Bioinf..

[57] Kaur G., Singh H.P., Batish D.R., Mahajan P., Kohli R.K., Rishi V. (2015). Exogenous Nitric oxide (NO) interferes with lead (pb)-induced toxicity by detoxifying reactive oxygen species in hydroponically grown wheat (*Triticum aestivum*) roots. PLoS ONE.

[58] Zanganeh R., Jamei R., Rahmani F. (2019). Role of salicylic acid and hydrogen sulfide in promoting lead stress tolerance and regulating free amino acid composition in *Zea mays* L.. Acta Physiol. Plant..

[59] Khan F., Hussain S., Tanveer M., Khan S., Hussain H.A., Iqbal B., Geng M. (2018). Coordinated effects of lead toxicity and nutrient deprivation on growth, oxidative status, and elemental composition of primed and non-primed rice seedlings. Environ. Sci. Pollut. Res..

[60] Fahr M., Laplaze L., Bendaou N., Hocher V., El Mzibri M., Bogusz D., Smouni A. (2013). Effect of lead on root growth. Front. Plant Sci..

[61] Ueno D., Koyama E., Yamaji N., Ma J.F. (2011). Physiological, genetic, and molecular characterization of a high-Cd-accumulating rice cultivar, Jarjan. J. Exp. Bot..

[62] Wang M., Chen L., Chen S., Ma Y. (2012). Alleviation of cadmium-induced root growth inhibition in crop seedlings by nanoparticles. Ecotoxicol. Environ. Saf..

[63] Blaylock M.J., Salt D.E., Dushenkov S., Zakharova O., Gussman C., Kapulnik Y., Ensley B.D., Raskin I. (1997). Enhanced accumulation of pb in indian mustard by soil-applied chelating agents. Environ. Sci. Technol..

[64] Siripornadulsil S., Traina S., Verma D.P.S., Sayre R.T. (2002). Molecular mechanisms of proline-mediated tolerance to toxic heavy metals in transgenic microalgae. Plant Cell.

[65] Hanikenne M., Talke I.N., Haydon M.J., Lanz C., Nolte A., Motte P., Kroymann J., Weigel D., Krämer U. (2008). Evolution of metal hyperaccumulation required cis-regulatory changes and triplication of *HMA4*. Nature.

[66] Yuan L., Yang S., Liu B., Zhang M., Wu K. (2012). Molecular characterization of a rice metal tolerance protein, *OsMTP1*. Plant Cell Rep..

[67] Derakhshani B., Jafary H., Maleki Zanjani B., Hasanpur K., Mishina K., Tanaka T., Kawahara Y., Oono Y. (2020). Combined QTL mapping and RNA-Seq profiling reveals candidate genes associated with cadmium tolerance in barley. PLoS ONE.

[68] Sundaria N., Singh M., Upreti P., Chauhan R.P., Jaiswal J.P., Kumar A. (2019). Seed priming with iron oxide nanoparticles triggers iron acquisition and biofortification in wheat (*Triticum aestivum* L.) grains. J. Plant Growth Regul..

[69] Gokak I.B., Taranath T.C. (2015). Morphological and biochemical responses of *Abelmoschus esculantus* (L.) Moench to zinc nanoparticles. Adv. Nat. Sci. Nanosci. Nanotechnol..

[70] Sharma S.S., Dietz K.-J. (2009). The relationship between metal toxicity and cellular redox imbalance. Trends Plant Sci..

[71] Liu N., Lin Z.-F., Lin G.-Z., Song L.-Y., Chen S.-W., Mo H., Peng C.-L. (2010). Lead and cadmium induced alterations of cellular functions in leaves of *Alocasia macrorrhiza* L. Schott. Ecotoxicol. Environ. Saf..

[72] Malecka A., Piechalak A., Tomaszewska B. (2009). Reactive oxygen species production and antioxidative defense system in pea root tissues treated with lead ions: The whole roots level. Acta Physiol. Plant..

[73] Kaur G., Singh H.P., Batish D.R., Kohli R.K. (2013). Lead (Pb)-induced biochemical and ultrastructural changes in wheat (*Triticum aestivum*) roots. Protoplasma.

[74] Holmström K.M., Finkel T. (2014). Cellular mechanisms and physiological consequences of redox-dependent signalling. Nat. Rev. Mol. Cell Biol..

[75] Xia C., Hong L., Yang Y., Yanping X., Xing H., Gang D. (2019). Protein changes in response to lead stress of lead-tolerant and lead-sensitive industrial hemp using SWATH technology. Genes.

[76] Dietz K.J., Baier M., Krämer U. (1999). Free radicals and reactive oxygen species as mediators of heavy metal toxicity in plants. Heavy Metal Stress in Plants.

[77] Meng J., Wang W.-X., Li L., Zhang G. (2018). Tissue-specific molecular and cellular toxicity of pb in the oyster (*Crassostrea gigas*): mRNA expression and physiological studies. Aquat. Toxicol..

