# Supplementary material for: Seed Priming with ZnO and Fe3O4 Nanoparticles Alleviate the Lead Toxicity in Basella alba L. through Reduced Lead Uptake and Regulation of ROS
Source: Plants (Basel). 2022 Aug 28;11(17):2227. doi: 10.3390/plants11172227 (PMC9460373; doi:10.3390/plants11172227)
Supplement: Supplementary file 1 [file plants-11-02227-s001.zip › plants-1861196-supplementary.pdf]

Supplementary Table S1. Effects of different Pb concentrations on seed germination, seedlings growth, seedling biomass, vigour indices and Growth Tolerance Index in 20 days old *B. alba* seedling

Lead (Pb) doses	Germination (%)	Root length (cm)	Shoot length (cm)	Seedling biomass (mg seedling^{-1})	Vigour Index-I	Vigour Index-II	GTI (%)		
							Root tissue	Shoot tissue	Seedling biomass
0 mM	71.0 ± 0.82 a	15.1 ± 0.24 a	11.6 ± 0.11 a	26.86 ± 0.08 a	1895.0 ± 12.1 a	1907.1 ± 3.7 a	-	-	-
2 mM	70.0 ± 0.74 a	13.6 ± 0.29 b	10.5 ± 0.18 b	23.47 ± 0.10 b	1689.1 ± 15.2 b	1642.9 ± 4.5 b	90.5	90.3	90.8
4 mM	65.0 ± 0.45 b	13.1 ± 0.16 c	9.4 ± 0.08 c	18.96 ± 0.09 c	1461.9 ± 9.5 c	1232.4 ± 6.5 c	86.7	81.2	73.3
6 mM	55.3 ± 0.55 c	9.4 ± 0.20 d	8.5 ± 0.19 d	15.07 ± 0.07 d	991.0 ± 9.9 d	833.4 ± 3.9 d	62.3	73.4	58.3
8 mM	49.3 ± 0.59 d	8.1 ± 0.18 e	7.9 ± 0.21 e	11.65 ± 0.11 e	786.3 ± 10.1 e	574.3 ± 7.5 e	53.7	67.6	45.1
10 mM	40.7 ± 0.49 e	5.6 ± 0.09 f	6.9 ± 0.10 f	7.89 ± 0.10 f	507.5 ± 13.2 f	321.1 ± 5.2 f	36.8	59.6	30.5
15 mM	31.3 ± 0.73 f	3.5 ± 0.12 g	5.3 ± 0.14 g	4.53 ± 0.11 g	276.4 ± 8.5 g	141.8 ± 5.0 g	23.3	45.8	17.5
20 mM	19.3 ± 0.68 g	1.2 ± 0.22 h	3.6 ± 0.19 h	2.32 ± 0.09 h	92.6 ± 10.3 h	44.8 ± 6.1 h	8.0	30.9	9.0

Values in the table are the mean of four replications ± standard error (SE). Different letters in each row are statistically significant at $p < 0.05$ level. Germination values are expressed in percentage and transformed to the respective angular (arc sin) values before subjecting them to statistical analysis.

Supplementary Table S2. Effects of seed priming with NPs and non-NPs on seed germination, seedlings growth, seedling biomass, vigour indices and Growth Tolerance Index (GTI) in 20 days old *B. alba* seedling

Treatments	Germination %	Root length (cm)	Shoot length (cm)	Seedling biomass (mg seedling^{-1})	Vigour Index-I	Vigour Index-II	GTI (%)		
							Root tissue	Shoot tissue	Seedling biomass
Control (0 mg.L^{-1} NP)	71.0 ± 0.82 cd	15.1 ± 0.24 d	11.6 ± 0.11 ef	26.86 ± 0.08 de	1895.0 ± 12.1 h	1907.1 ± 3.7 e	-	-	-
50 mg.L^{-1} ZnNPs	74.7 ± 0.77 bc	15.2 ± 0.08 d	11.3 ± 0.08 f	26.90 ± 0.21 de	1976.6 ± 18.2 fgh	2009.4 ± 5.1 de	100.5	97.3	100.1
100 mg.L^{-1} ZnNPs	76.3 ± 0.92 b	15.9 ± 0.09 c	12.0 ± 0.12 de	27.12 ± 0.23 d	2127.2 ± 14.2 cd	2069.3 ± 9.6 d	105.5	103.1	101.0
200 mg.L^{-1} ZnNPs	82.3 ± 0.62 a	17.3 ± 0.11 b	13.9 ± 0.15 b	30.42 ± 0.19 b	2566.1 ± 8.5 b	2503.6 ± 3.3 b	114.9	119.4	113.3
300 mg.L^{-1} ZnNPs	75.7 ± 0.88 b	14.1 ± 0.12 e	11.2 ± 0.09 f	26.52 ± 0.18 e	1915.2 ± 11.0 gh	2007.6 ± 8.4 de	93.6	96.4	98.7
500 mg.L^{-1} ZnNPs	67.3 ± 0.51 d	12.1 ± 0.08 f	8.7 ± 0.07 g	21.45 ± 0.09 g	1401.2 ± 13.1 i	1443.6 ± 7.2 f	80.3	75.0	79.9
50 mg.L^{-1} FeNPs	75.7 ± 0.42 b	15.2 ± 0.18 d	11.4 ± 0.15 f	26.76 ± 0.24 de	2017.4 ± 12.9 efg	2025.7 ± 7.9 de	101.1	98.3	99.6
100 mg.L^{-1} FeNPs	77.3 ± 0.55 b	15.8 ± 0.16 c	12.7 ± 0.11 c	28.45 ± 0.13 c	2199.2 ± 11.8 c	2199.2 ± 4.6 c	104.7	109.0	105.9
200 mg.L^{-1} FeNPs	84.3 ± 0.67 a	18.2 ± 0.10 a	15.0 ± 0.10 a	31.24 ± 0.17 a	2796.2 ± 10.5 a	2633.5 ± 5.1 a	120.9	128.7	116.3
300 mg.L^{-1} FeNPs	76.0 ± 0.70 b	14.8 ± 0.13 d	12.3 ± 0.13 cd	25.25 ± 0.09 f	2059.6 ± 9.4 def	1919.0 ± 6.3 e	98.2	105.9	94.0
500 mg.L^{-1} FeNPs	68.7 ± 0.89d	10.3 ± 0.15 g	8.3 ± 0.14 g	20.14 ± 0.14 h	1281.9 ± 9.8 j	1383.6 ± 5.9 f	68.6	71.6	75.0
ddH$_2$O (Hydro-primed)	74.3 ± 0.53 bc	15.9 ± 0.09 c	12.6 ± 0.08 c	28.14 ± 0.10 c	2116.1 ± 10.2 cde	2090.8 ± 4.8 cd	105.2	108.6	104.8
200 mg.L^{-1} ZnSO$_4$.7H$_2$O	74.8 ± 0.66 bc	16.1 ± 0.07 c	12.4 ± 0.05 cd	28.51 ± 0.14 d	2131.5 ± 13.6 cd	2057.0 ± 4.6 d	106.6	106.9	106.1
200 mg.L^{-1} FeSO$_4$.7H$_2$O	75.3 ± 0.49 b	16.6 ± 010 bc	13.2 ± 0.08 c	29.10 ± 0.19 c	2243.9 ± 7.7 c	2115.9 ± 5.8 cd	109.9	113.8	108.3

Values in the table are the mean of four replications ± standard error (SE). Different letters in each row are statistically significant at $p < 0.05$ level. Germination values are expressed in percentage and transformed to the respective angular (arc sin) values before subjecting them to statistical analysis.
